# Inhibition of constitutive and cxc-chemokine-induced NF-*κ*B activity potentiates ansamycin-based HSP90-inhibitor cytotoxicity in castrate-resistant prostate cancer cells

**DOI:** 10.1038/sj.bjc.6605356

**Published:** 2009-10-06

**Authors:** A Seaton, P J Maxwell, A Hill, R Gallagher, J Pettigrew, R H Wilson, D J J Waugh

**Affiliations:** 1 Centre for Cancer Research and Cell Biology, Queen's University Belfast, 97 Lisburn Road, Belfast BT9 7BL, Northern Ireland

**Keywords:** prostate cancer, CXC-chemokines, CXCL8, NF-*κ*B, Hsp90 inhibitors

## Abstract

**Background:**

We determined how CXC-chemokine signalling and necrosis factor-*κ*B (NF-*κ*B) activity affected heat-shock protein 90 (Hsp90) inhibitor (geldanamycin (GA) and 17-allylamino-demethoxygeldanamycin (17-AAG)) cytotoxicity in castrate-resistant prostate cancer (CRPC).

**Methods:**

Geldanamycin and 17-AAG toxicity, together with the CXCR2 antagonist AZ10397767 or NF-*κ*B inhibitor BAY11-7082, was assessed by 3-(4, 5-Dimethylthiazol-2-yl)-2, 5-diphenyltetrazolium bromide assay in two CRPC lines, DU145 and PC3. Flow cytometry quantified apoptotic or necrosis profiles. Necrosis factor-*κ*B activity was determined by luciferase readouts or indirectly by quantitative PCR and ELISA-based determination of CXCL8 expression.

**Results:**

Geldanamycin and 17-AAG reduced PC3 and DU145 cell viability, although PC3 cells were less sensitive. Addition of AZ10397767 increased GA (e.g., PC3 IC_20_: from 1.67±0.4 to 0.18±0.2 nM) and 17-AAG (PC3 IC_20_: 43.7±7.8 to 0.64±1.8 nM) potency in PC3 but not DU145 cells. Similarly, BAY11-7082 increased the potency of 17-AAG in PC3 but not in DU145 cells, correlating with the elevated constitutive NF-*κ*B activity in PC3 cells. AZ10397767 increased 17-AAG-induced apoptosis and necrosis and decreased NF-*κ*B activity/CXCL8 expression in 17-AAG-treated PC3 cells.

**Conclusion:**

Ansamycin cytotoxicity is enhanced by inhibiting NF-*κ*B activity and/or CXC-chemokine signalling in CRPC cells. Detecting and/or inhibiting NF-*κ*B activity may aid the selection and treatment response of CRPC patients to Hsp90 inhibitors.

There has been considerable interest in the clinical application of inhibitors of heat-shock protein 90 (Hsp90), particularly in cancer therapeutics (Smith *et al*, 1998; Maloney and Workman, 2002; Isaacs *et al*, 2003; Workman, 2004; Solit and Rosen, 2006). Heat-shock protein 90 is a molecular chaperone protein, the function of which is to ensure the correct folding of a well-defined group of client proteins, many of which have been shown to have a role in tumourigenesis (Jolly and Morimoto, 2000). Depending on its conformation, Hsp90 recruits various co-chaperones to assemble two distinct complexes, one of which favours proteasome-dependent degradation of the client protein and one that favours stabilisation (Meyer *et al*, 2003). Several classes of Hsp90 inhibitors have been developed, but the majority of research to date has been conducted exploiting the ansamycin class of inhibitors, of which the natural product geldanamycin (GA) and its synthetic analogue, 17-allylamino-demethoxygeldanamycin (17-AAG), are the best-known molecules. Geldanamycin and 17-AAG stabilise an Hsp90 conformation that recruits co-chaperones to target Hsp90 client proteins for proteasomal degradation (Mimnaugh *et al*, 1996; Roe *et al*, 1999). Both inhibitors have shown preclinical activity in a range of human cancer models, but GA was too toxic in animal testing to progress into the clinic. Furthermore, recent phase I clinical trials with 17-AAG or its primary metabolite, 17-dimethylaminoethylamino-17-demethoxygeldananmycin (17-DMAG), have determined tolerable toxicity with these agents and reported prolonged stable disease in treated patients (Maloney and Workman, 2002; Sausville *et al*, 2003; Pacey *et al*, 2006; Ramanthan *et al*, 2007; Solit *et al*, 2007; Pacey *et al*, 2009).

17-Allylamino-demethoxygeldanamycin has been shown to exhibit activity in a number of prostate cancer (CaP) models (Solit *et al*, 2002; Solit and Rosen, 2003). Although many CaP patients initially respond to therapy, a significant proportion of patients relapse after radiation therapy and/or androgen ablation therapy, leading to the acquisition of a castration-resistant, metastatic disease. Treatment options for patients with castration-resistant, metastatic CaP are very limited, principally because of the intrinsic chemoresistance acquired by cells during disease progression on therapy. Therefore, the potential to use Hsp90 inhibitors in this disease setting is an exciting and important prospect; treatment responses to 17-DMAG have been reported in two patients with castrate-resistant prostate cancer (CRPC) for 16 and 33 months in a phase I trial (Pacey *et al*, 2009).

CXCL8 (also known as interleukin-8) is a CXC chemokine that modulates the function of target cells through activation of two receptors, CXCR1 and CXCR2, which have a similar affinity for CXCL8 but otherwise exhibit markedly different selectivity for related CXC chemokines (Waugh and Wilson, 2008). Increased expression of CXCL8, CXCR1 and CXCR2 genes has been detected in CaP tissue through the exploitation of *in situ* hybridisation (Uehara *et al*, 2005) and/or immunohistochemistry (Murphy *et al*, 2005; Huang *et al*, 2005a), whereas increased levels of CXCL8 have been detected in the serum of patients with pathologically confirmed CaP (Veltri *et al*, 1999). Therefore, CaP cells and tumour stromal cells are subject to an increased CXC-chemokine signalling stimulus within the tumour microenvironment, underpinning a range of tumour-promoting functions (Waugh and Wilson, 2008; Waugh *et al*, 2008). In the context of CaP, CXCL8 expression correlates with increased angiogenesis, tumourigenicity and lymph node metastasis *in vivo* (Inoue *et al*, 2000; Kim *et al*, 2001). We, along with others, have shown that CXCL8 signalling contributes to the transition of a disease to a castration-resistant state (Araki *et al*, 2007; Seaton *et al*, 2008). Furthermore, we have shown that CXC-chemokine signalling (including CXCL8) modulates the sensitivity of CaP cells to environmental stresses such as hypoxia and chemotherapy agents, including the DNA-damage agents oxaliplatin and etoposide (Maxwell *et al*, 2007; Wilson *et al*, 2008a).

17-Allylamino-demethoxygeldanamycin has been shown to enhance the cytotoxicity of several conventional chemotherapeutic drugs in representative cell-based models of colorectal cancer and CaP (Munster *et al*, 2001; Rakitina *et al*, 2003; Vasilevskaya and O’Dwyer, 2005). The capacity of 17-AAG to reduce the activation of NF-*κ*B has been proposed as a potential mode of action in sensitising cancer cells to oxaliplatin and 5-FU (Rakitina *et al*, 2003). However, the relevance of constitutive or ligand-induced NF-*κ*B activity in directly regulating the sensitivity of cancer cells to Hsp90 inhibitors has not been studied extensively. Increased NF-*κ*B activity has been reported in CaP cell lines and tissue (Fradet *et al*, 2004; Sweeney *et al*, 2004; Li *et al*, 2005; Huang *et al*, 2005b). Therefore, as Hsp90 inhibitors hold promise in treating CRPC, the objective of this study was to determine whether the intrinsic levels of NF-*κ*B activity and/or constitutive CXC-chemokine signalling may correlate with and modulate the efficacy of Hsp90-targeted therapies in CRPC cells.

## Materials and methods

### Cell culture

PC3 and DU145 cells were sourced and cultured as previously described (MacManus *et al*, 2007; Maxwell *et al*, 2007).

### Chemical and reagents

Chemicals were sourced from Sigma-Aldrich (St Louis, MO, USA), unless otherwise stated. BAY11-7082 was purchased from Calbiochem (La Jolla, CA, USA). AZ10397767 ((1*R*)-5-[[(3-chloro-2-fluorophenyl)methyl]thio]-7-[[2-hydroxy-1-methylethyl]amino]thiazolo[4,5-*d*]pyrimidin-2(3*H*)-one) was kindly provided by Dr Simon Barry and Dr David Blakey (AstraZeneca, Alderley Park, Cheshire, UK) (Walters *et al*, 2007).

### 3,3′-dihexyloxacarbocyanine iodide and propidium iodide staining

Cells were seeded into 24-well plates (1 × 10^5^ cells per well) in RPMI 1640 medium and allowed to attach overnight. The CXCR2 antagonist AZ10397767 (20 nM) was added to the cells in conjunction with 17-AAG (1 nM or 1 *μ*M). Plates were incubated in a humidified chamber at 37°C with 5% CO_2_ for 72 h; thereafter, 3,3′-dihexyloxacarbocyanine iodide (DiOC_6_(3)) (40 nM) was added and the plates were incubated for a further 15 min. Propidium iodide (PI) (20 *μ*g ml^−1^) was added, plates were returned to the incubator for 10 min and cells were collected (floating and adherent cells removed by trypsin). The cells were washed once in PBS and re-suspended in 500 *μ*l PBS and analysed by flow cytometry (Beckman Coulter, Buckinghamshire, UK). Plots of FL1 (DiOC6(3)) *vs* FL4 (PI) were divided into quadrants to assess the percentage of cells living, cells undergoing necrosis and cells at early and late stages of apoptosis (Lecoeur *et al*, 2008).

### Quantitative real-time PCR

RNA was harvested and prepared for analysis by quantitative real-time (qPCR) as previously described (Wilson *et al*, 2008a). The cDNA (50 ng) was mixed with primers (2 nM), sterile water and SYBR Green PCR mastermix (Finnzymes Diagnostics, Espoo, Finland). The primer sequences were as follows: 18s: forward: 5′-CATTCGTATTGCGCCGCT-3′, reverse: 5′-CGACGGTATCTGATCGTC-3′; HSP90: forward: 5′-AAAGCCCATCTGGACCAGAA-3′, reverse: 5′-AGCACGTCGTGGGACAAATA-3′. Primers for CXCL8 have been described previously. Standard cycling procedures were used with an annealing temperature of 55°C and an amplification temperature of 95°C. Specific amplicon formation with each primer pair was confirmed by melt curve analysis, and gene expression was quantified relative to an 18 s housekeeping gene.

### Immunoblotting

Protein was prepared, resolved and blotted as described previously (Murphy *et al*, 2005; MacManus *et al*, 2007). Membranes were washed in Tris-buffered saline/0.1% Tween-20 (TBS-T), then blocked for 1 h at room temperature in 5% bovine serum albumin/TBS-T (BSA/TBS-T). Expression of Hsp90 was detected using an anti-human Hsp90 Ab (Cell Signaling Technology Inc, Danvars, MA, USA) at 1 : 1000 dilution in 5% BSA/TBS-T. The membranes were washed thrice in TBS-T and then incubated with a rabbit horse-radish peroxidase (HRP)-labelled secondary antibody (GE Healthcare UK Ltd, Buckinghamshire, UK). After three washes in TBS-T, the bands were detected using enhanced chemiluminescence (ECL plus reagents, Amersham Biosciences, Amersham, UK). Membranes were re-probed to ensure equal loading with GAPDH antibody (Biogenesis, Dorset, UK).

### 3-(4, 5-Dimethylthiazol-2-yl)-2, 5-diphenyltetrazolium bromide cell viability assays

Cells were seeded into 96-well plates (3 × 10^3^ cells per well) in RPMI 1640 medium and allowed to attach overnight. Serial dilutions of 17-AAG or GA were added to the cells alone or in combination with AZ10397767 (20 nM). Plates were incubated in a humidified chamber at 37°C with 5% CO_2_ for 72 h; thereafter, 50 *μ*l 3-(4, 5-Dimethylthiazol-2-yl)-2, 5-diphenyltetrazolium bromide (MTT) (2 mg ml^−1^) was added and the plates were returned to the incubator for 4 h. Medium and any non-metabolised MTT was aspirated from the wells and formazan crystals were dissolved in 100 *μ*l dimethyl sulphoxide. Absorbance was read at 570 nm using a microplate reader (Molecular Devices, Wokingham, UK). Data were plotted using a nonlinear regression curve fitting algorithm (GraphPad Prism 4.0, La Jolla, CA, USA).

### Luciferase reporter assays

The detection of NF-*κ*B-driven transcriptional activity in unstimulated cells was determined using the NF-*κ*B-LUC-pGL3 plasmid as previously described (Wilson *et al*, 2008a).

### Statistical analysis

Student's *t*-test was used to compare means where appropriate. All tests were two-tailed.

## Results

### Inhibition of constitutive CXC-chemokine signalling confers cell-specific ansamycin-induced cytotoxicity in CRPC cells

**Figure 1:**
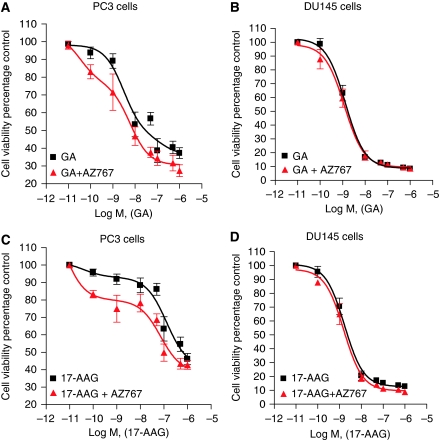
Inhibition of CXC-chemokine signalling sensitises PC3 but not DU145 cells to ansamycin antibiotics, geldanamycin (GA) and 17-AAG. Survival curves representing cell viability in PC3 (**A** and **C**) cells and DU145 (**B** and **D**) cells, as measured by MTT assay, conducted after a 72 h exposure to increasing concentrations of either GA or 17-AAG. The consequence of inhibiting CXC-chemokine signalling on the effect of these compounds on cell viability was determined by co-administration of the CXCR2 antagonist AZ10397767 (AZ767) at a final concentration of 20 nM. Values are expressed as the mean±s.e.m. of five separate experiments.

Our experiments were conducted on two representative models of CRPC, the PC3 and DU145 cell lines. 3-(4, 5 -Dimethylthiazol-2-yl)-2, 5-diphenyltetrazolium bromide assays demonstrated that PC3 cells were differentially sensitive to GA and 17-AAG, with GA exhibiting greater activity (Figure 1A and B; Table 1). In contrast, DU145 cells were equally sensitive to these two agents (Figure 1C and D; Table 1). Interestingly, the DU145 cell line was also found to be markedly more sensitive to these ansamycin-based compounds in comparison with PC3 cells; GA was 17-fold more potent in DU145 cells, whereas the potency of 17-AAG was 275-fold greater in DU145 cells compared with PC3 cells.

We previously showed that inhibition of CXCR2 signalling can increase the sensitivity of CaP cells to conventional cytotoxic chemotherapeutic agents and death receptor ligands (Wilson *et al*, 2008a, 2008b). Therefore, we determined whether blockade of constitutive CXC-chemokine signalling using the CXCR2 receptor antagonist, AZ10397767, sensitised PC3 and DU145 cells to GA and 17-AAG. AZ10397767 was administered to cells concurrently at a concentration of 20 nM, exploiting its selectivity to block CXCR2 signalling. Addition of AZ10397767 to PC3 cells enhanced the cytotoxicity of both Hsp90 inhibitors, increasing the potency (IC_50_) of GA and of 17-AAG by three-fold and 3.5-fold, respectively (Table 1). However, analysis of data using nonlinear regression illustrated that the augmentation of 17-AAG cytotoxicity in PC3 cells was more striking at lower concentrations, wherein the calculated IC_20_ value was observed to have increased 68-fold after co-administration of AZ10397767 with 17-AAG. In marked contrast, the inhibition of CXCR2 signalling did not increase the cytotoxicity of either GA or 17-AAG in DU145 cells.

### Inhibition of CXCR2 signalling potentiates 17-AAG-cytotoxicity through promotion of increased apoptosis and necrosis in CRPC cells

**Figure 2:**
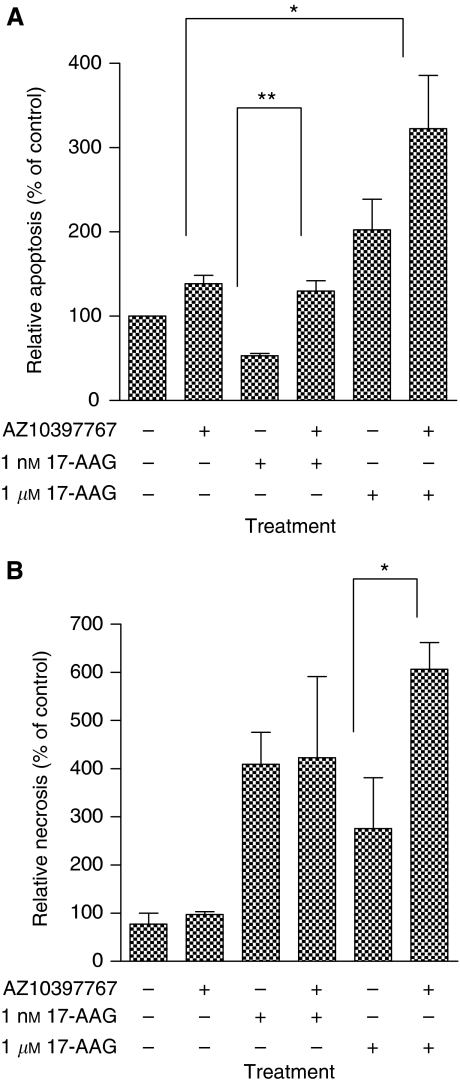
Inhibition of CXC-chemokine signalling increases the ability of 17-AAG to induce both apoptosis and necrosis in PC3 cells. (**A**) Bar graphs representing flow cytometric analysis of PC3 cells stained with DiOC_6_(3) and propidium iodide to assess the consequence of inhibition of CXCR2 receptor signalling to the induction of apoptosis (**A**) and necrosis (**B**) by 72 h exposure to 17-AAG (1 nM or 1 *μ*M). This was determined by co-administration of the CXCR2 antagonist AZ10397767 at a final concentration of 20 nM. Values are expressed as the mean±s.e.m. of five separate experiments, ^*^P<0.05; ^**^P<0.01.

Experiments were conducted to determine the mechanism through which the inhibition of CXCR2 signalling may increase the sensitivity of PC3 cells to 17-AAG. Cultured PC3 cells demonstrate low levels of apoptosis (3–4% of total cells) and necrosis (1–2% of total cell population) under normal culture conditions. Administration of AZ10397767 on its own had a minimal effect on the basal level of necrosis detected in PC3 cells, whereas it had a modest effect in increasing the level of apoptosis (Figure 2A and B). Administration of 1 nM 17-AAG alone failed to induce apoptosis (Figure 2A) but selectively increased the level of necrosis detected in PC3 cells (Figure 2B). Co-administration of AZ10397767 with low-dose 17-AAG increased the level of apoptosis (*P*<0.01) but not necrosis in PC3 cells. The level of apoptosis detected from the combination of both drugs was similar to the effect of administering AZ10397767 alone. Administration of high concentrations of 17-AAG revealed the induction of both apoptosis and necrosis in PC3 cells. The levels of Hsp90-inhibitor-induced apoptosis and necrosis were increased when AZ10397767 was co-administered with 1 *μ*M 17-AAG; the increase in necrosis after addition of AZ10397767 was significant (*P*<0.05), whereas the increase in apoptosis bordered on statistical significance. These results suggest that this Hsp90-targeted agent is cytotoxic to PC3 cells by promoting both necrotic and apoptotic cell death, mechanisms that seem to be selectively induced in response to low and high concentrations of 17-AAG, respectively. Our results suggest that inhibition of CXCR2 signalling increases the efficacy of 17-AAG-induced apoptosis across the entire concentration–response curve, whereas it increases necrosis only at higher concentrations of 17-AAG.

### Inhibition of constitutive NF-*κ*B activity enhances sensitivity of PC3 cells to 17-AAG

**Figure 3:**
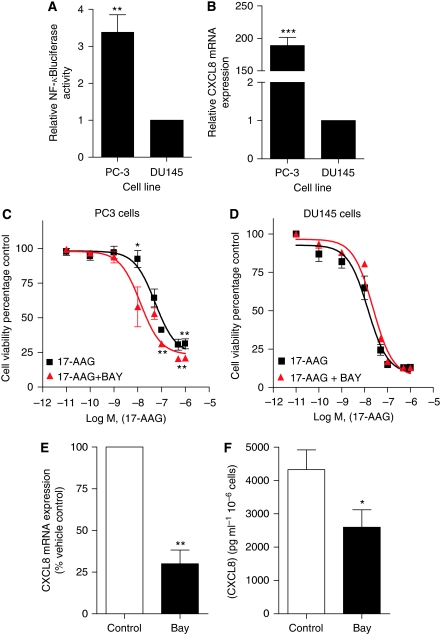
Inhibition of NF-*κ*B signalling sensitises PC3 but not DU145 cells to 17-AAG. (**A**) Bar graph comparing the NF-*κ*B transcriptional activity in PC3 and DU145 cells. This was determined using an NF-*κ*B-driven luciferase activity in transiently transfected PC3 and DU145 cells. Cells were co-transfected with either NF-*κ*B luciferase reporter (2 *μ*g) or pGL3 basic vector (2 *μ*g), and with *Renilla* luciferase reporter (0.02 *μ*g), and incubated for 72 h before collection and analysis. Transfection efficiencies were adjusted relative to *Renilla* readouts and luciferase activities were normalised to pGL3 values. Values are expressed as the mean±s.e.m. of six separate experiments, ^**^P<0.01. (**B**) Bar graph comparing IL-8 expression levels in PC3 and DU145 cells. Real-time PCR analysis of IL-8 mRNA transcript levels was used for this comparison. Values are expressed as the mean±s.e.m. of five separate experiments, ^***^P<0.001. (**C** and **D**) Survival curves representing cell viability in PC3 cells (**C**) and DU145 cells (**D**), as measured by MTT assay, conducted after a 72 h exposure to increasing concentrations of 17-AAG. The consequence of inhibiting NF-*κ*B signalling on the effect of these compounds on cell viability was determined by co-administration of the NF-*κ*B antagonist BAY-11-7082 (BAY) at a final concentration of 10 nM. Values are expressed as the mean±s.e.m. of five separate experiments, ^*^P>0.05; ^**^P<0.01. (**E**) Bar graph illustrating relative changes in CXCL8 mRNA transcript levels in PC3 cells after treatment with BAY-11-7082 for 6 h. Values are expressed as the mean±s.e.m. of five separate experiments, ^**^P<0.01. (**F**) Bar graph illustrating the change in CXCL8 secretion detected by specific ELISA after treatment with BAY-11-7082 for 6 h. Values are expressed as the mean±s.e.m. of four separate experiments, ^*^P<0.05.

Initial experiments were designed to confirm the level of constitutive NF-*κ*B activity in DU145 and PC3 cells. Using an NF-*κ*B-specific luciferase reporter assay, we determined a 3.2-fold higher luciferase activity in transfected PC3 cells relative to transfected DU145 cells (*P*<0.01) (Figure 3A). Furthermore, qPCR-based determination of CXCL8 mRNA confirmed a marked elevation in the expression of this NF-*κ*B-transcriptional target in PC3 cells relative to DU145 cells (*P*<0.001) (Figure 3B). We previously confirmed an equivalent expression of CXCR1 and CXCR2 receptors in these cell lines (Murphy *et al*, 2005).

3-(4, 5-Dimethylthiazol-2-yl)-2, 5-diphenyltetrazolium bromide assays were established to determine the relationship of constitutive NF-*κ*B activity and the differential sensitivity of PC3 and DU145 cells to 17-AAG. Constitutive NF-*κ*B activity was inhibited using a final concentration of 0.1 *μ*M BAY11-7082, chosen on the basis that this concentration of drug exhibited limited toxicity of its own to these CRPC cells. As before, increasing concentrations of 17-AAG resulted in a concentration-dependent decrease in PC3 cell viability. At each concentration used, the cytotoxicity of 17-AAG was enhanced in the presence of 0.1 *μ*M BAY11-7082 (Figure 3C), promoting a leftwards and parallel displacement of the concentration–response curve and equating it to a 4.1-fold increase in IC_50_ for 17-AAG in these cells. As seen before with AZ10397767, the presence of BAY11-7082 was shown to sensitise cells to low concentrations of 17-AAG (e.g., 10 nM 17-AAG). In contrast, administration of BAY11-7082 had no effect in potentiating the cytotoxicity of 17-AAG in DU145 cells (Figure 3D). This supports our hypothesis that constitutive NF-*κ*B activity may account in part for the reduced sensitivity of PC3 cells to Hsp90 inhibitors.

The effect of adding BAY11-7082 on the endogenous levels of CXCL8 in CRPC cells was also confirmed by qPCR and ELISA. Administration of BAY11-7082 was shown to reduce the endogenous mRNA transcript level of vehicle-treated controls for CXCL8 to 30±8.3% (*P*<0.01) within 6 h (Figure 3E). Similarly, the rate of CXCL8 secretion from PC3 cells was similarly decreased after a 6 h exposure to BAY11-7082 (*P*<0.05) (Figure 3F). Therefore, these experiments establish a link between elevated constitutive NF-*κ*B activity and increased endogenous CXCL8 expression in the PC3 cell line.

**Figure 4:**
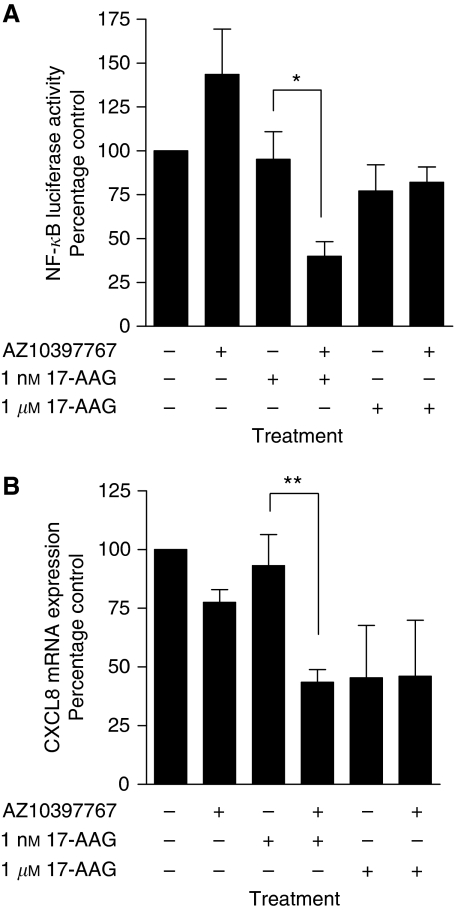
Effects of drug treatment on NF-*κ*B activity in PC3 cells. (**A**) Bar graph illustrating the effects of 17-AAG and/or AZ10397767 administration on NF-*κ*B transcriptional activity in PC3 cells. Drug-induced changes in NF-*κ*B-driven luciferase activity were measured and adjusted, as previously described (legend to Figure 3), 24 h after addition of drugs. Values are expressed as the mean±s.e.m. of five separate experiments, ^**^P<0.01. (**B**) Bar graph representing real-time PCR analysis of CXCL8 mRNA transcript levels in PC3 cells after 24 h exposure to 17-AAG (1 nM or 1 *μ*M). Cells were pre-treated for 4 h with 20 nM AZ10397767 or vehicle control to assess the effect on cellular IL-8 expression, used here as a readout of constitutive NF-*κ*B transcription. Values are expressed as the mean±s.e.m. of five separate experiments, ^*^P<0.05, ^**^P<0.01.

Further experiments were conducted to characterise how the co-administration of AZ10397767 with 17-AAG effected NF-*κ*B transcriptional activity in PC3 cells. Using an NF-*κ*B luciferase reporter assay, administration of AZ10397767 for 24 h was shown to induce a small but not statistically significant increase in NF-*κ*B transcriptional activity in PC3 cells. In contrast, neither concentration (1 nM or 1 *μ*M) increased the activity of this transcription factor. However, co-administration of AZ10397767 with 1 nM 17-AAG was observed to decrease NF-*κ*B transcriptional activity in PC3 cells (*P*<0.05) (Figure 4A). This result was further supported by analysis of CXCL8 mRNA expression, used in this context as a readout of NF-*κ*B activity (again determined 24 h after the addition of drugs). By itself, the administration of AZ10397767 was shown to reduce the expression of CXCL8 mRNA to 73% of that determined in control cells (Figure 4B). Treatment with 1 nM 17-AAG and 1 *μ*M 17-AAG promoted concentration-dependent decreases in the constitutive CXCL8 mRNA levels determined in cells. The addition of AZ10397767, together with 1 nM 17-AAG, had a pronounced effect in reducing CXCL8 mRNA expression (*P*<0.01). No further decrease in CXCL8 mRNA levels was observed by the addition of AZ10397767 with the higher concentration of 17-AAG. This suggests that the addition of AZ10397767 to low concentrations of 17-AAG results in the maximal repression of NF-*κ*B activity that can be exerted by these compounds in PC3 cells.

### CXC-chemokine signalling induces Hsp90 expression in CRPC cells through increased Hsp90 gene transcription

**Figure 5:**
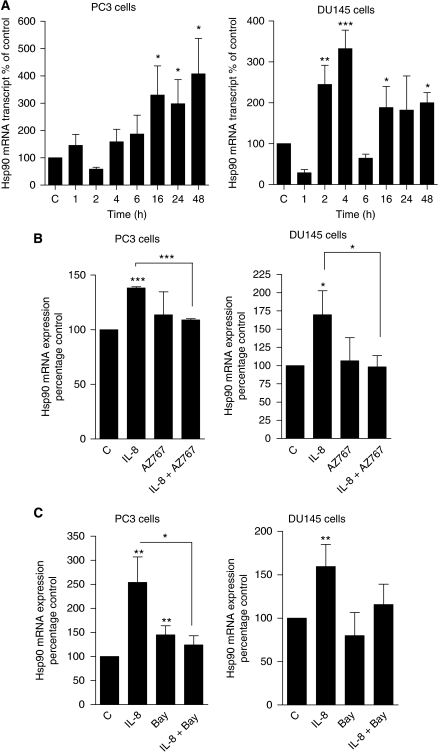
Interleukin-8 induces Hsp90 gene transcription in CRPC cells. (**A**) Bar graphs representing real-time PCR analysis of Hsp90 mRNA transcript levels in PC3 (left panel) and DU145 (right panel) cells after stimulation with 3 nM rh-IL-8 for the indicated times. Values are expressed as the mean±s.e.m. of four and five independent experiments, respectively; ^*^P<0.05; ^**^P<0.01; ^***^P<0.001. (**B** and **C**) Bar graphs illustrating qPCR data for Hsp90 transcript levels in PC3 cells (left panels) and DU145 cells (right panels). Cells were pre-treated for 4 h with either the CXCR2 antagonist AZ10397767 at a final concentration of 20 nM (**B**) or BAY-11-7082 at a final concentration of 5 *μ*M (**C**), before stimulation with 3 nM rh-IL-8 for 24 h. Data shown are shown as mean±s.e.m., calculated from six independent experiments, ^*^P<0.05; ^**^P<0.01; ^***^P<0.001.

Our data indicate that elevated NF-*κ*B activity and CXCL8 expression correlate with reduced sensitivity of PC3 cells to 17-AAG and that inhibition of either NF-*κ*B or CXCR2 signalling increases the sensitivity of PC3 cells to this Hsp90 inhibitor. The induction of CXC-chemokine signalling is a well-characterised response of cells to stress. Therefore, we used qPCR assays and immunoblotting experiments to determine whether CXCL8 signalling regulated Hsp90 expression in CRPC cells. Addition of exogenous CXCL8, at a concentration of 3 nM, to both PC3 and DU145 cells resulted in a time-dependent increase in the expression of Hsp90 mRNA transcripts in both cell lines (Figure 5A, left and right panels). Although Hsp90 mRNA levels were increased in both cell lines, there was an apparent difference in the temporal regulation of Hsp90 transcripts between the two cell lines; stimulation with CXCL8 was shown to induce rapid increases in the transcription of the Hsp90 gene (2–4 h) in DU145 cells, whereas significant changes were only detected in PC3 cells 16 h after treatment with CXCL8.

The link of CXCR2 and NF-*κ*B signalling in underpinning CXCL8-promoted transcription of the Hsp90 gene in the two CRPC cell lines was investigated in a further series of experiments. Cells were stimulated with 3 nM CXCL8 in the absence and presence of 20 nM AZ10397767 for 24 h before analysis of Hsp90 mRNA transcript levels. Administration of AZ10397767 attenuated the CXCL8-induced increase in Hsp90 mRNA transcript levels in both PC3 and DU145 cells (Figure 5B, left and right panel). In addition, attenuation of NF-*κ*B activity, effected by the co-administration of 5 *μ*M BAY11-7082, blocked the CXCL8-promoted increases in Hsp90 mRNA transcript levels in PC3 cells and DU145 cells (Figure 5C, left and right panel).

**Figure 6:**
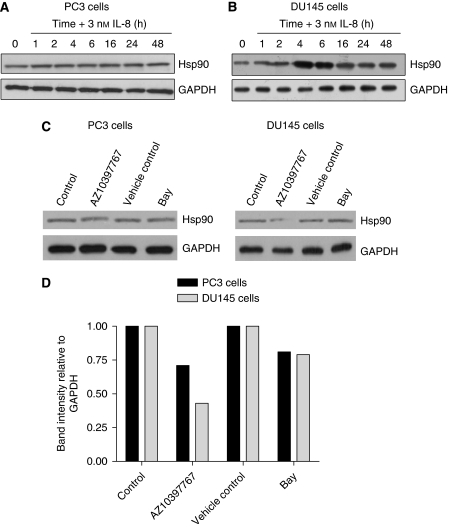
Interleukin-8 signalling regulates Hsp90 expression in DU145 cells. Immunoblots illustrating the expression of Hsp90 in cell lysates extracted from (**A**) PC3 and (**B**) DU145 cells stimulated with 3 nM rh-IL-8 for indicated times. Equal protein loading was confirmed by re-probing the membranes for GAPDH. (**C**) Immunoblot detecting levels of Hsp90 expression in the absence and presence of the CXCR2 inhibitor AZ10397767 (20 nM) and BAY11-7082 (5 *μ*M), administered to both PC3 and DU145 cells for 16 h. (**D**) Bar graph presenting densitometry analysis of the immunoblots presented in (**C**) above. The ratio of Hsp90/GAPDH expression is presented as a percentage of the ratio detected in controls.

Administration of rh-CXCL8 was also shown to result in an increased Hsp90 expression at the protein level in DU145 cells, with peak increases in expression detected 4 and 6 h after stimulation (Figure 6B). In contrast, addition of CXCL8 failed to potentiate the protein expression of Hsp90 in PC3 cells under these experimental conditions (Figure 6A). PC3 cells exhibit higher levels of constitutive CXCL8 expression than do DU145 cells (Figure 3B), which may explain our inability to observe CXCL8-induced potentiation of Hsp90 protein expression in these cells. To test this, AZ10397767 and BAY11-7082 were administered to PC3 and DU145 cells for 16 h to determine whether the inhibition of constitutive CXC chemokine or NF-*κ*B signalling in these cells altered Hsp90 expression. Densitometry analysis of immunoblots confirmed that, relative to internal controls, addition of AZ10397767 reduced the endogenous expression of Hsp90 to 70 and 45% in PC3 and DU145 cells, respectively, compared with that detected in vehicle-treated controls. Addition of the NF-*κ*B inhibitor reduced endogenous expression of Hsp90 to ∼80% of untreated controls in either cell line (Figure 6C and D).

## Discussion

Our results identify a central role of NF-*κ*B activity in modulating the response of CRPC cells to treatment with ansamycin-based Hsp90 inhibitors. Comparison of the relative potencies of GA- and 17-AAG cytotoxicity in the two CRPC cell lines reveals a reduced sensitivity of PC3 cells to each of the Hsp90-targeted therapeutic compounds, correlating with the elevated level of constitutive NF-*κ*B transcriptional activity detected in these cells. Pharmacological inhibition of this constitutive NF-*κ*B activity in these cells using BAY11-7082 resulted in an increased sensitisation of cells to 17-AAG. In contrast, addition of BAY11-7082 to the more sensitive DU145 cells had no effect on the potency of 17-AAG, consistent with the reduced activity of NF-*κ*B detected in this cell line. Previous pre-clinical and clinical observations have suggested that Hsp90 inhibitors may be most effective when used as ‘chemomodulators’, exploiting their reported capacity to reduce NF-*κ*B activity to sensitise metastatic cancer cells to chemotherapeutic and biological agents such as oxaliplatin and TRAIL (Rakitina *et al*, 2003). The data presented in this paper indicate that the efficacy of Hsp90 inhibitors themselves is enhanced when combined with direct inhibitors of NF-*κ*B activity.

The relevance of NF-*κ*B activity in determining the reduced sensitivity to ansamycin-based inhibitors of Hsp90 may be two-fold. First, NF-*κ*B activity is known to confer a cell survival advantage through regulation of antiapoptotic gene expression. Thus, silencing of NF-*κ*B activity will reduce the expression of known antiapoptotic proteins and may concurrently reduce the transcription and expression of other Hsp90-client proteins, many of which are critical to the survival of cells with oncogenic transformation. Our studies also show that the inhibition of NF-*κ*B activity reduces chemokine-induced increases in mRNA transcript levels for Hsp90 in the PC3 cell line, indicating that NF-*κ*B contributes to the transcriptional regulation of the Hsp90 gene in CRPC cells. Consequently, the elevated NF-*κ*B activity in PC3 cells may define a multifaceted resistance that functionally antagonises Hsp90-directed therapeutic agents, initially through a promotion of compensatory survival signaling, and then through underpinning the increased expression of Hsp90 in these cells.

We have shown that exposure to chemotherapy agents induces NF-*κ*B-driven CXC-chemokine signalling in CRPC cells (Wilson *et al*, 2008a, 2008b). These studies also characterise that CXC-chemokine signalling is coupled to NF-*κ*B transcriptional activity and thus potentiates chemotherapy-induced activation of this transcription factor and downstream antiapoptotic gene expression (Wilson *et al*, 2008a, 2008b). Inhibition of this stress-induced CXC-chemokine signalling results in the sensitisation of CaP cells to chemotherapy and chemotherapy-induced apoptosis (Maxwell *et al*, 2007; Wilson *et al*, 2008a). In this study, we have shown that pharmacological inhibition of CXCR2 signalling results in a cell-dependent sensitisation of PC3 cells but not DU145 cells to the Hsp90 inhibitors, GA and 17-AAG. The capacity of AZ10397767 to increase ansamycin cytotoxicity in PC3 cells, especially at lower concentrations of 17-AAG, seems to be related to the reduced levels of constitutive NF-*κ*B activity detected in PC3 cells receiving these compounds in combination. This was exemplified by direct measurements of NF-*κ*B luciferase activity and by using analysis of CXCL8 gene transcription as a readout of NF-*κ*B activity; co-administration of AZ10397767 with 1 nM 17-AAG attenuated transcriptional activity and reduced the level of constitutive CXCL8 mRNA transcripts detected relative to that in untreated cells or those treated with 17-AAG alone. Accordingly, we propose on the basis of this and previously published studies that the blockade of CXCR2 signalling using AZ10397767 (i) acts initially to inhibit the CXC-chemokine-promoted activation of NF-*κ*B and (ii) as a consequence of that action, will promote a secondary response, suppressing NF-*κ*B-promoted regulation of CXCL8 and anti-apoptotic gene expression. Consequently, the capacity to perturb NF-*κ*B activity and selectively increase apoptosis levels provides a primary mechanism of action that explains the capacity of AZ10397767 to sensitise PC3 cells to Hsp90 inhibitors, especially at low concentrations of ansamycin compounds. The lower level of NF-*κ*B activity detected in DU145 cells is consistent with the inability of either the CXCR2 receptor antagonist or the NF-*κ*B inhibitor to potentiate the response of these cells to 17-AAG or GA.

We have also shown that CXC-chemokine signalling modulates the transcription of the Hsp90 gene in CRPC cells through a CXCR2-, NF-*κ*B-mediated pathway. Interestingly, CXCL8-promoted Hsp90 gene regulation was biphasic in DU145 cells, suggesting that the regulation of this gene may be promoted through the action of multiple and temporally regulated transcription factors in this cell line. Administration of BAY11-7082 reversed the CXC-chemokine-promoted induction of Hsp90 mRNA transcripts in both cell lines, measured 24 h after the addition of stimulus, indicating that this later phase of transcriptional regulation is probably dependent on chemokine-promoted NF-*κ*B activity. However, the transcription factor(s) underpinning the early increase in Hsp90 gene transcription in DU145 cells remain to be determined. Although an increased Hsp90 gene transcription was evident from qPCR analysis, immunoblotting experiments illustrated only quantitative increases in Hsp90 expression in CXCL8-stimulated DU145 cells. Transient blockade of constitutive CXC-chemokine signalling using the CXCR2 antagonist did reveal decreases in endogenous Hsp90 expression in both cells, confirming the importance of this receptor in regulating the transcription and expression of this chaperone protein. Therefore, as the CXCR2 antagonist reduces Hsp90 expression in both PC3 and DU145 cells, these data suggest that the sensitising effect of AZ10397767 on the cytotoxicity of Hsp90 inhibitors is more likely explained by the inhibition of constitutive NF-*κ*B activity, as opposed to a direct modulation of Hsp90 expression.

Understanding the cellular response to Hsp90-targeted therapeutics is complex and multifaceted, given the extensive list of client proteins and pathways that are regulated by this protein. The underlying genetic dysfunction of tumour cells is likely to have a marked impact in determining the response of cells to Hsp90 inhibitors. For example, a functional retinoblastoma (Rb) protein has previously been shown to be required to promote ansamycin-mediated G1 arrest and apoptosis in breast cancer cells. Cells with defective Rb were subsequently shown to undergo mitotic arrest before the induction of apoptosis (Munster *et al*, 2001). In this study, we showed that cells with increased NF-*κ*B activity are less sensitive to ansamycins and that inhibiting the constitutive activity of this transcription factor can sensitise CRPCs to 17-AAG. This observation may be important for consideration in future strategies aimed at exploiting Hsp90 inhibitors in the treatment of metastatic CaP, as nuclear distribution and increased NF-*κ*B activity have been characterised in a significant proportion of advanced CaP cases (Fradet *et al*, 2004; Sweeney *et al*, 2004). It is already widely accepted that future clinical development of Hsp90 inhibitors is likely to see these agents being exploited in combination therapy as opposed to their use in monotherapy. Therefore, co-administration with agents that directly perturb NF-*κ*B activity may provide a useful strategy to sensitise tumours to Hsp90-targeted therapy. Furthermore, analysis of diagnostic biopsy tissue for evidence of elevated NF-*κ*B activity may prove to be a useful test to predict those tumours that are unlikely to show clinical response to ansamycin-based inhibitors.

In conclusion, these studies add to a series of studies illustrating the linkage of CXC-chemokine signalling to the development of chemoresistance in CaP cells. We have shown that constitutive NF-*κ*B and/or CXC-chemokine signalling decreases the efficacy of Hsp90 inhibitors in a CRPC cell line that exhibits elevated activity and expression of these entities. This raises the prospect that elevated CXC-chemokine expression detected in patient serum and/or NF-*κ*B activity in diagnostic biopsy specimens may serve as predictive biomarkers of tumour response to the Hsp90 inhibitors that are used in treating this disease.

## Figures and Tables

**Table 1:** Potency of Hsp90-inhibitor cytotoxicity to CRPC cell lines in the absence and presence of the CXCR2 receptor antagonist AZ10397767

		**IC_50_** **values (nM)**		**IC_20_ values (nM)**	
**Cell line**	**Compound**	**Hsp90 inh alone**	**Hsp90 inh+AZ10397767**	**Hsp90 inh alone**	**Hsp90 inh+AZ10397767**
PC3	GA	29.9±4.2	8.34±2.1	1.67±0.4	0.18±0.2
	17-AAG	640±125	185±67	43.7±7.8	0.64±1.8
DU145	GA	1.69±0.08	1.42±0.18	0.42±0.11	0.31±0.09
	17-AAG	2.31±0.4	1.81±0.5	0.52±0.2	0.38±0.2

Abbreviations: GA=geldanamycin: 17-AAG=17-allylamino-demethoxygeldanamycin.
